# Long-Term Deflection Prediction from Computer Vision-Measured Data History for High-Speed Railway Bridges

**DOI:** 10.3390/s18051488

**Published:** 2018-05-09

**Authors:** Jaebeom Lee, Kyoung-Chan Lee, Young-Joo Lee

**Affiliations:** 1 School of Urban and Environmental Engineering, Ulsan National Institute of Science and Technology (UNIST), Ulsan 44919, Korea; jblee@unist.ac.kr; 2 Advanced Railroad Civil Engineering Division, Korea Railroad Research Institute, Uiwang 16105, Korea

**Keywords:** railway bridge, vertical deflection, probabilistic prediction, Gaussian process, training data

## Abstract

Management of the vertical long-term deflection of a high-speed railway bridge is a crucial factor to guarantee traffic safety and passenger comfort. Therefore, there have been efforts to predict the vertical deflection of a railway bridge based on physics-based models representing various influential factors to vertical deflection such as concrete creep and shrinkage. However, it is not an easy task because the vertical deflection of a railway bridge generally involves several sources of uncertainty. This paper proposes a probabilistic method that employs a Gaussian process to construct a model to predict the vertical deflection of a railway bridge based on actual vision-based measurement and temperature. To deal with the sources of uncertainty which may cause prediction errors, a Gaussian process is modeled with multiple kernels and hyperparameters. Once the hyperparameters are identified through the Gaussian process regression using training data, the proposed method provides a 95% prediction interval as well as a predictive mean about the vertical deflection of the bridge. The proposed method is applied to an arch bridge under operation for high-speed trains in South Korea. The analysis results obtained from the proposed method show good agreement with the actual measurement data on the vertical deflection of the example bridge, and the prediction results can be utilized for decision-making on railway bridge maintenance.

## 1. Introduction

Rail transport is one of the essential infrastructure systems that support human life and carry a large amount of freight and many passengers. Various research efforts have been devoted to develop faster and safer train systems, and as a result, high-speed trains, such as the Shinkansen in Japan, Inter City Express (ICE) in Germany, Train à Grande Vitesse (TGV) in France, and Korea Train eXpress (KTX) in Korea are in operation worldwide. In addition to the development of high-speed trains, the importance of building and managing railway structures that allow high-speed trains to operate safely has been recognized. Especially, managing the long-term vertical deflection of a bridge is essential for the traffic safety and passenger comfort of the high-speed trains.

In the case of a railway bridge, vertical deflection is one of the important indicators for inspecting its overall safety and for reporting abnormalities [1]. Moreover, it significantly influences the running stability of a train vehicle, particularly when traveling at high speed [2]. Therefore, keeping the vertical deflection of a railway bridge below a certain level is crucial for high-speed trains. Indeed, several standards, such as UIC CODE 518 OR (2009), Design Guide for Steel Railway Bridges of UK (2004), and Guideline of Track Maintenance of Korea Rail Network Authority (2016), regulate guides to check the safety and comfort of rail traffic in terms of an acceptable vertical deflection at mid-span [3,4,5].

Thus, many researchers have been trying to develop efficient sensors and methods to measure the vertical deflection of railway bridges. A conventional contact type sensor is a linear variable differential transformer (LVDT), which uses electric signals and has been used widely for structural health monitoring [6]. However, it requires installing a device at a measurement point on a robust platform of a bridge, which is often unfeasible or impractical [7]. The accelerometer, which is another widely used contact type sensor, does not require such installation. However, deflection results based on accelerometers may have numerical errors, because the deflection calculation requires the double integral of the measured acceleration [7]. To overcome the limitations of contact type sensors, various non-contact type sensors, such as global positioning systems (GPS), laser Doppler vibrometers (LDV), Radio Detection and Ranging (RADAR), and vision-based systems, have also been developed. Although GPS, LDV, and RADAR can measure the vertical deflection of a bridge accurately, they are relatively expensive [8,9,10,11].

On the other hand, vision-based systems, which use video images to measure the bridge deflection, are widely used [7,12,13] because these systems are (1) relatively affordable; (2) sufficiently precise for measuring the vertical deflection of bridges; (3) easy to install; (4) equipped with small-sized and lightweight hardware; and (5) easy to upgrade by introducing a high-frame-rate camera [14,15]. Vision-based systems have been advanced in several aspects: target or non-target approaches, feature detection, and coordinate transform algorithms [12]. In order to detect features, the target-based approach utilizes a target marker which is attached to a structure [12,13,16]; whereas the non-target-based approach uses features directly from a structure [7,17]. For example, Lee et al. (2017) developed a target-based system [12], and Fukuda et al. (2013) suggested a system without the installation of a target panel [7]. A more comprehensive review on vision-based systems is provided by Lee et al. [12]. Despite the recent advances in vision-based systems, the measurement results are also known to be affected by several factors such as wind-shaking and camera location, which may produce measurement errors.

Meanwhile, research efforts have attempted to predict the vertical deflection of railway bridges, and many of the previous studies focused on the derivation of physics-based models about the vertical deflection [18,19,20]. In particular, prestressed concrete girders, which are often used for railway bridges, are known to be deflected for various reasons including temperature changes, concrete creep, shrinkage, and train loads. Thus, Guo et al. (2010) attempted to construct an equation to mathematically represent the vertical deflection of a railway bridge by considering these physical phenomena, and the actual measurement results were mainly used to validate the equation [18]. Similarly, Khan and Kim (2012) presented finite element model-based prediction on long-term beam deflection which fitted to the experimental measurement [21]. Although the prediction on the vertical deflection in these studies showed decent agreement with the actual measurement data, the physics-based models need to be sophisticated when factors which were not considered in the models need to be addressed [22]. In addition, most of the previous studies did not take into account the uncertainty factors associated with railway bridges and could not give a probabilistic prediction on their vertical deflection [23].

Therefore, this study proposes a probabilistic method employing a Gaussian process (GP) to predict the vertical deflection of a railway bridge based on actual vision-based measurement data and temperature. The vision-based measurements of an example bridge were obtained using the method of Kim and Kim (2014), in which a Digital Image Correlation (DIC) technique is introduced to estimate the vertical deflection of a target [24,25,26,27]. To deal with the sources of uncertainty which may cause prediction errors, the GP is modeled with multiple kernels and hyperparameters. Once the hyperparameters are identified through GP regression using training data, the proposed method provides the 95% prediction interval as well as the predictive mean of the vertical deflection of a railway bridge.

## 2. Proposed Method

### 2.1. Gaussian Process Regression

This research aims to construct a probabilistic model to predict the vertical deflection of a railway bridge, based on actual measurement data on vertical deflection and temperature. When a prediction model is developed, in general, there are several sources of uncertainty related to the model such as model misspecification, limited data size, and inherent variability, which are known to introduce errors of bias, model variance, and noise into the model [28,29]. Using the prediction error decomposition proposed by Geman et al. (1992), the expectation of a squared prediction error can be decomposed into these errors [30]. As model variance and bias occur because of misspecification of the model or limited data size, these values are reducible. Meanwhile, noise cannot be reduced because it occurs owing to inherent variability, and it is an irreducible error [31]. When constructing a prediction model, it is thus important to introduce an appropriate mathematical model capable of addressing these three types of errors.

In this respect, many researchers have constructed probabilistic prediction models for their engineering problems and tested these models by setting a confidence interval (CI) or a prediction interval (PI) [32]. The term CI refers to an interval concerning only the reducible error (i.e., bias and model variance). Meanwhile, the PI considers the irreducible error (i.e., noise) in addition to the reducible error. This study aims to develop a probabilistic prediction model which can deal with both of the reducible and irreducible errors and provide prediction results with a PI about the vertical deflection of high-speed railway bridges.

To build a probabilistic prediction model, in this study, a GP is introduced. It is a machine learning-based method building a flexible Bayesian model [33], and it requires regression analysis to identify appropriate GP parameters based on training data through optimization. The details of GPs can be found in Rasmussen and Williams (2006) [34], and are briefly explained in this paper.

GPs are based on the Gaussian (i.e., normal) distribution, and a GP can be modeled by a regression mean and a covariance matrix, which often requires high computational costs. Despite their high costs, GPs have been widely utilized in many applications owing to their theoretical simplicity and great performance to build a probabilistic model [34].

GPs are based on the following assumptions: (1) every data point is associated with a normally distributed random variable; and (2) the finite collection of these random variables is multivariate normally distributed, also explained as jointly normal. The multivariate normality is often introduced to explain a set of correlated random data which cluster around the mean, and it simplifies the related calculations with its definition that any sub-vector of the multivariate random variables is again a Gaussian random vector [35]. For example, when one has a noisy training dataset **D** from *N_D_* times of measurement, which consists of a training input matrix **X** and a training output vector **y**, the dataset can be expressed as

(1) $$D=\left\{\left(X,y\right)\right\}=\left\{\left(x_{ij},y_{i}\right)|i=1,\ldots ,N_{D};j=1,\ldots ,N_{x}\right\},$$

where *x_ij_* is an element of the training input matrix **X**, which is constructed from *N_D_* times of observation for *N_x_* variables, and *y_i_* is an element in the training output vector **y** whose size is *N_D_* by 1. Then, the two assumptions of GPs enable the prediction of the Gaussian mean and variance of the output.

For a prediction purpose, when a test input matrix **X_∗_** is introduced to estimate the corresponding unknown test output vector ${\hat{f}}_{*}\left(\cdot \right)$, the combined vector of the known training output **y** and the unknown test output ${\hat{f}}_{*}\left(\cdot \right)$ is expressed as the multivariate Gaussian variables:

(2) $$\left(\begin{array}{c} y\\ {\hat{f}}_{*}\left(X_{*},\hat{\theta }\right) \end{array}\right)=N\left(O,\Sigma \right)=N\left(O,\left[\begin{array}{cc} K+\sigma_{\mathrm{noise}}^{2}I & K_{*}\\ K_{*}^{T} & K_{**}+\sigma_{\mathrm{noise}}^{2}I \end{array}\right]\right),$$

where *N* denotes the multivariate Gaussian distribution, **O** is the zero matrix, which is often introduced as a prior mean function for numerical simplicity [29,33,34], **∑** is the symmetric and positive semidefinite covariance matrix, **K** is the covariance matrix of the training input matrix **X**, **K_∗_** is the covariance matrix between training (**X**) and test (**X_∗_**) inputs, **K_∗∗_** is the covariance matrix of the test input matrix **X_∗_**, and $\sigma_{\mathrm{noise}}^{2}$ is the variance of noise. In the covariance matrix **∑**, noise is assumed to be independent and identically normally distributed. Because the noise exists in the test data as well as in the training data, the noise variance $\sigma_{\mathrm{noise}}^{2}$ is added to all diagonal terms in the covariance matrix **∑**. When the sizes of the training input matrix and the test input are *N_D_* by *N_X_* and *N_P_* by *N_X_*, respectively, the sizes of the covariance matrices **K**, **K_∗_**, **K_∗∗_**, and **∑** are *N_D_* by *N_D_*, *N_D_* by *N_P_*, *N_P_* by *N_P_*, and (*N_D_* + *N_P_*) by (*N_D_* + *N_P_*), respectively:

(3) $$\begin{array}{l} \sum =\left[\begin{array}{cc} K+\sigma_{\mathrm{noise}}^{2}I & K_{*}\\ K_{*}^{T} & K_{**}+\sigma_{\mathrm{noise}}^{2}I \end{array}\right]=\\ \left[\begin{array}{cc} \left[\begin{array}{ccc} k\left(x_{1},x_{1}\right) & \cdots & k\left(x_{1},x_{N_{D}}\right)\\ \vdots & \ddots & \vdots \\ k\left(x_{N_{D}},x_{1}\right) & \cdots & k\left(x_{N_{D}},x_{N_{D}}\right) \end{array}\right] & \left[\begin{array}{ccc} k\left(x_{1},x_{1}^{*}\right) & \cdots & k\left(x_{1},x_{N_{P}}^{*}\right)\\ \vdots & \ddots & \vdots \\ k\left(x_{N_{D}},x_{1}^{*}\right) & \cdots & k\left(x_{N_{D}},x_{N_{P}}^{*}\right) \end{array}\right]\\ {\left[\begin{array}{ccc} k\left(x_{1},x_{1}^{*}\right) & \cdots & k\left(x_{1},x_{N_{P}}^{*}\right)\\ \vdots & \ddots & \vdots \\ k\left(x_{N_{D}},x_{1}^{*}\right) & \cdots & k\left(x_{N_{D}},x_{N_{P}}^{*}\right) \end{array}\right]}^{T} & \left[\begin{array}{ccc} k\left(x_{1}^{*},x_{1}^{*}\right) & \cdots & k\left(x_{1}^{*},x_{N_{P}}^{*}\right)\\ \vdots & \ddots & \vdots \\ k\left(x_{N_{P}}^{*},x_{1}^{*}\right) & \cdots & k\left(x_{N_{P}}^{*},x_{N_{P}}^{*}\right) \end{array}\right] \end{array}\right]=\\ \left[\begin{array}{cc} \left[\begin{array}{ccc} \sigma_{x_{1}}^{2} & \cdots & \sigma_{x_{1}}\sigma_{x_{N_{D}}}\rho_{x_{1}x_{N_{D}}}\\ \vdots & \ddots & \vdots \\ \sigma_{x_{N_{D}}}\sigma_{x_{1}}\rho_{x_{N_{D}}x_{1}} & \cdots & \sigma_{x_{N_{D}}}^{2} \end{array}\right] & \left[\begin{array}{ccc} \sigma_{x_{1}}\sigma_{x_{1}^{*}}\rho_{x_{1}x_{1}^{*}} & \cdots & \sigma_{x_{1}}\sigma_{x_{N_{P}}^{*}}\rho_{x_{1}x_{N_{P}}^{*}}\\ \vdots & \ddots & \vdots \\ \sigma_{x_{N_{D}}}\sigma_{x_{1}^{*}}\rho_{x_{N_{D}}x_{1}^{*}} & \cdots & \sigma_{x_{N_{D}}}\sigma_{x_{N_{P}}^{*}}\rho_{x_{N_{D}}x_{N_{P}}^{*}} \end{array}\right]\\ {\left[\begin{array}{ccc} \sigma_{x_{1}}\sigma_{x_{1}^{*}}\rho_{x_{1}x_{1}^{*}} & \cdots & \sigma_{x_{1}}\sigma_{x_{N_{P}}^{*}}\rho_{x_{1}x_{N_{P}}^{*}}\\ \vdots & \ddots & \vdots \\ \sigma_{x_{N_{D}}}\sigma_{x_{1}^{*}}\rho_{x_{N_{D}}x_{1}^{*}} & \cdots & \sigma_{x_{N_{D}}}\sigma_{x_{N_{P}}^{*}}\rho_{x_{N_{D}}x_{N_{P}}^{*}} \end{array}\right]}^{T} & \left[\begin{array}{ccc} \sigma_{x_{1}^{*}}^{2} & \cdots & \sigma_{x_{1}^{*}}\sigma_{x_{{}_{N_{P}}}^{*}}\rho_{x_{1}^{*}x_{{}_{N_{P}}}^{*}}\\ \vdots & \ddots & \vdots \\ \sigma_{x_{{}_{N_{P}}}^{*}}\sigma_{x_{1}^{*}}\rho_{x_{{}_{N_{P}}}^{*}x_{1}^{*}} & \cdots & \sigma_{x_{{}_{N_{P}}}^{*}}^{2} \end{array}\right] \end{array}\right] \end{array},$$

where **x** and **x^∗^** are the 1 by *N_X_* vectors of training and test inputs, *k*(·) is an element termed the kernel in the covariance matrix **∑**, $\rho_{x_{a}x_{b}}$ is the correlation between **x***_a_* and **x***_b_*, and $\sigma_{x_{a}}^{2}$ is the variance of **x***_a_*.

In Equation (3), the covariance is often modeled by a kernel function associated with the Euclidian distance between two inputs [34], and one popular choice of the kernel considering correlation is the squared exponential (SE) kernel shown below:

(4) $$k_{SE}(x_{a},x_{b})=\sigma_{f}^{2}\exp\left(-\frac{1}{2}\frac{\sqrt{\sum_{i=1}^{N_{x}}{\left[x_{a}\left(1,i\right)-x_{b}\left(1,i\right)\right]}^{2}}}{l^{2}}\right),$$

where $\sigma_{f}^{2}$ is the variance hyperparameter of inputs which controls the vertical scale of the function change and *l* is a length-scale hyperparameter that is associated with the horizontal scale of the function change [29].

However, as shown in Equation (3), the covariance matrix **∑** requires additional variance terms for its diagonal terms (i.e., $\sigma_{\mathrm{noise}}^{2}I$). This study introduces the Kronecker delta function [34]:

(5) $$k_{var}(x_{a},x_{b})=\sigma_{f}^{2}\cdot \delta (x_{a},x_{b}),$$

where *δ*(*x_i_*,*x_j_*) is the Kronecker delta function, which becomes one when the two inputs are the same, and zero when they are different.

In other words, *k_SE_* and *k_var_* need to be introduced to construct the covariance matrix. Based on this idea, a new kernel was proposed by summing up existing kernels without any loss of properties as kernel [34]:

(6) $$k(x_{a},x_{b})={\hat{\theta }}_{0}\exp\left(-\frac{1}{2}\frac{\sqrt{\sum_{i=1}^{N_{x}}{\left[x_{a}\left(1,i\right)-x_{b}\left(1,i\right)\right]}^{2}}}{{\hat{\theta }}_{1}^{2}}\right)+{\hat{\theta }}_{2}\cdot \delta (x_{a},x_{b})+{\hat{\theta }}_{3}$$

where ${\hat{\theta }}_{0}$ and ${\hat{\theta }}_{1}$ are hyperparameters for non-diagonal terms, ${\hat{\theta }}_{2}$ is a hyperparameter to control the variance in diagonal terms, and ${\hat{\theta }}_{3}$ is a hyperparameter to control the level of the overall values in the covariance matrix. The kernel in Equation (6) was applied to several previous studies [36,37,38], and it is introduced to this study for building the covariance matrix in Equation (3).

A GP is sensitive to these hyperparameters; thus, for the purpose of prediction, the determination of these parameters based on the given measurement data through optimization is important to build an accurate prediction model. For the task, the concept of maximum likelihood is often introduced [28].

Regarding Equations (1) and (2), the likelihood of observing the training output vector **y** given the training input **X** can be expressed as a conditional probability using the multivariate normal distribution as follows:

(7) $$p(y|X)=\frac{1}{{\left(2\pi \right)}^{\frac{N_{D}}{2}}\cdot {\left|\Sigma \right|}^{\frac{1}{2}}}\cdot \exp\left(-\frac{1}{2}y^{T}\Sigma^{-1}y\right).$$

For numerical convenience, the natural logarithm is introduced for conditional probability and is multiplied by minus one:

(8) $$L=-\ln p(y|X)=\frac{1}{2}y^{T}{\left(K+\sigma_{\mathrm{noise}}^{2}I\right)}^{-1}y+\frac{1}{2}\ln\left|K+\sigma_{\mathrm{noise}}^{2}I\right|+\frac{N_{D}}{2}\ln\left(2\pi \right),$$

where *L* is the log-likelihood function. The three terms on the right in Equation (8) represent the three errors in the prediction model described in Section 2.1 (i.e., the bias, model variance, and noise).

Then, the best hyperparameters ${\hat{\theta }}_{best}$ can be determined through optimization to minimize the sum of these errors (i.e., the log-likelihood function *L* in Equation (8)):

(9) $${\hat{\theta }}_{best}=\underset{\hat{\theta }}{\arg\min}(L).$$

Since the conditional probability was multiplied by minus one as shown in Equation (8), ${\hat{\theta }}_{best}$ obtained from the minimization problem in Equation (9) become the parameters which maximize the likelihood of observing the training output vector **y** given the training input **X**.

Once the hyperparameters are identified using Equation (9), the kernel in Equation (6) is constructed. Then, the optimal covariance matrix in Equation (3) is composed and the mean and the variance vectors of the test inputs can be calculated by the property of the multivariate Gaussian distribution. Based on the derivation by Muirhead (2009), given test input **X_∗_**, the conditional multivariate Gaussian distributions of the test outputs ${\hat{f}}_{*}\left(\cdot \right)$ are estimated as follows [39]:

(10) $${\hat{f}}_{*}\left(\cdot \right)|y,X,X_{*}\sim N\left({Κ}_{*}^{T}{\left(Κ+\sigma_{\mathrm{noise}}^{2}I\right)}^{-1}y,K_{**}-K_{*}^{T}{\left(K+\sigma_{\mathrm{noise}}^{2}I\right)}^{-1}K_{*}+\sigma_{\mathrm{noise}}^{2}I\right).$$

Using Equation (10), the predictive mean and the PI can be estimated.

### 2.2. Performance Assessment of Predictive Mean and Prediction Interval

First, for the performance assessment of the predictive mean, a prediction error index is defined. In this regard, the root-mean-square error (RMSE) has been widely used to quantify the prediction error [40]:

(11) $$\mathrm{RMSE}=\sqrt{\frac{\sum_{i=1}^{N_{p}}{\left(\hat{f}(x_{i})-y(x_{i})\right)}^{2}}{N_{p}}},$$

where $\hat{f}\left(x_{i}\right)$ is the predictive mean at the test input **x***_i_* and *y*(**x***_i_*) is the actual measurement. Although the RMSE is a great test indicator of the predictive mean, it is also known to have limitations for a fluctuating dataset, which can be easily found in the dataset of vertical deflection of a railway bridge. A fluctuating dataset and the associated prediction, which is not overfitted and follows the fluctuating trend, could have a large RMSE due to the inherent variability underlying the data. For assessing how well the prediction follows the trend of the fluctuating dataset, the mean-error (ME) is widely used as a good alternative indicator [41,42,43]:

(12) $$\mathrm{ME}=\frac{\sum_{i=1}^{N_{p}}\left(\hat{f}(x_{i})-y(x_{i})\right)}{N_{p}}.$$

Second, for the performance assessment of a PI, its coverage probability needs to be assessed [32]. The coverage probability is generally defined by the percentage of actual measurement values covered by a PI. A popular index is the PI coverage probability (PICP):

(13) $$\mathrm{PICP}=\frac{1}{N_{p}}\sum_{i=1}^{N_{p}}C_{i},$$

where *C_i_* is the Boolean value which can be evaluated as:

(14) $$C_{i}=\{\begin{array}{cc} 1, & y_{i}\in \left[L_{i},U_{i}\right]\\ 0, & y_{i}\notin \left[L_{i},U_{i}\right] \end{array},$$

where *y_i_* is the *i*th measurement data, *L_i_* is the lower bound, and *U_i_* is the upper bound of the PI. When all the measurement values are located in the PI, PICP becomes 1. In practice, the PI is built with the nominal confidence of (1 − α)%, which is known as the PI nominal confidence (PINC), and a good PI should have a similar PICP with the given confidence level. In this respect, the average coverage error (ACE) was defined with PICP and PINC as a performance measure of the PI [44]:

(15) ACE=PINC−PICP.

In this study, RMSE and ME are used to assess the performance of the predictive mean, and ACE is introduced to estimate the performance of the PI.

## 3. Application Example

### 3.1. Example Bridge: Eonyang Arch Bridge

**Figure 1:**
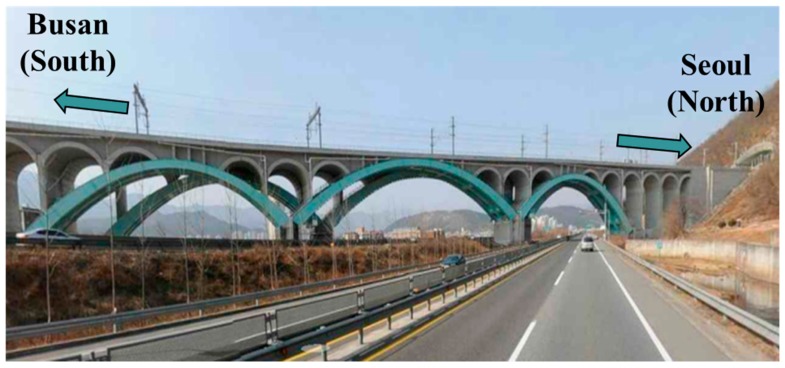
Eonyang Arch Bridge.

The proposed method was applied to Eonyang Arch Bridge, which is a set of arch bridges for high-speed trains and is located in Ulsan, South Korea. As shown in Figure 1, two twin bridges are neighbored for north- and southbound traffic, respectively. The northbound bridge was used as the test target structure in this study, and is shown in Figure 1.

**Figure 2:**
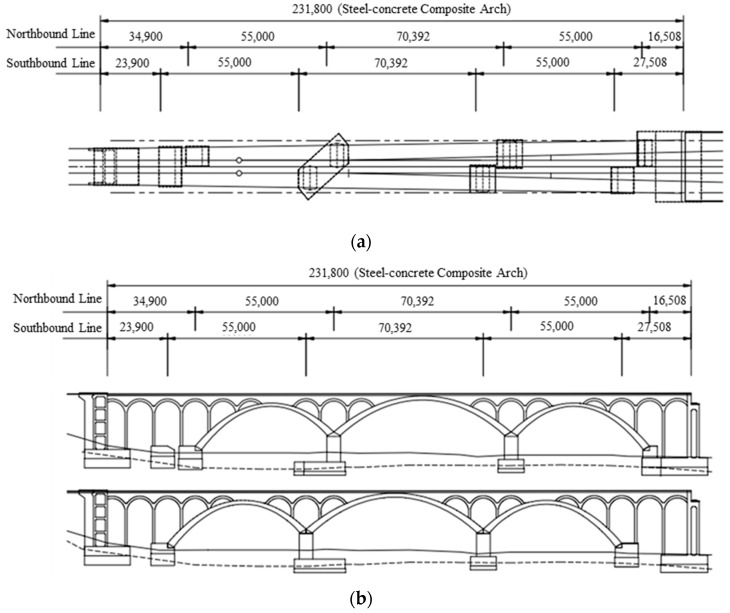
Design drawings of Eonyang Arch Bridge: (**a**) plan view; (**b**) front view.

Eonyang Arch Bridge was constructed in 2009 as a steel–concrete composite arch bridge for high-speed trains, the Korea Train Express (KTX), and Figure 2 shows its design drawings in the plan and front views. The bridge has a total length of 231.8 m, a center span of 70.4 m, a height of 10 m. Its substructure consists of three main arches rigidly connected to the foundation and several small arches over the main arch ribs. This unique structure consisting of main and small arches is designed to reduce the additional axial force of continuously welded rail on the bridge, and to provide a uniform level of stiffness over the entire bridge to enhance the comfort of passengers traveling in high-speed trains. Since the arch ribs are rigidly connected to the foundation, they deform vertically because of temperature change. The effect of this vertical deformation was evaluated for traffic safety and passenger comfort [45] and showed that upward deformation can adversely affect passenger comfort in hot weather over 40 degrees Celsius.

### 3.2. Measurement

**Figure 3:**
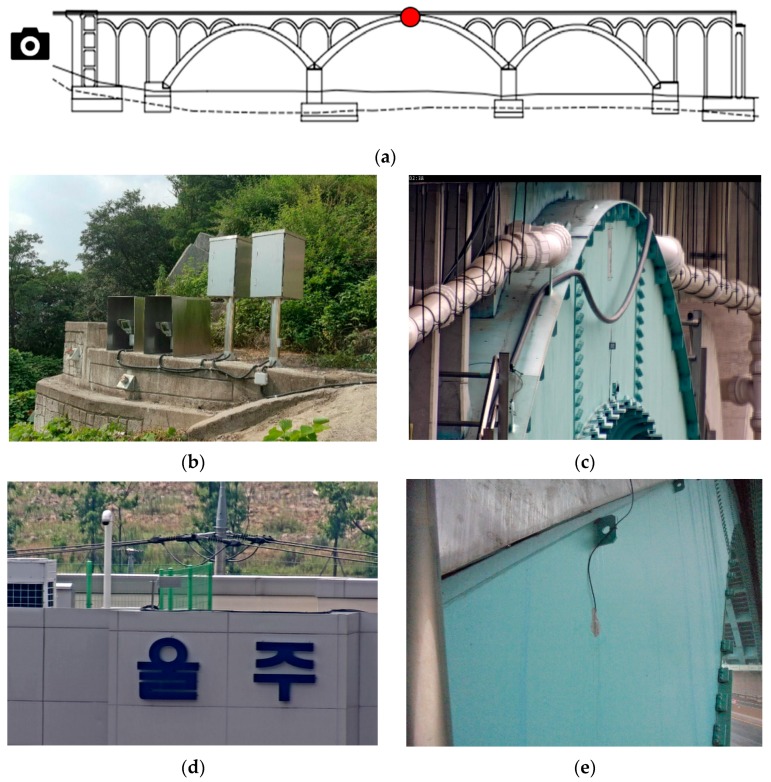
(**a**) Measurement location; (**b**) two video cameras; (**c**) bridge target video camera view for deflection measurement; (**d**) reference target video camera view for camera movement correction; (**e**) Resistance Temperature Detector (RTD) and its installation.

The vertical deflection of Eonyang Arch Bridge and temperature were measured by the vision-based system and the Resistance Temperature Detector (RTD), respectively. First, the deflection at the center of the mid-span was measured as shown in Figure 3a. Two HDTV video cameras were installed at a slope at the end of the bridge. The cameras have image sensors with progressive scan RGB CMOS 1/2” with 1920 × 1080 pixels and zoom lenses with focal lengths of 10–350 mm with a 35× zooming feature. The cameras were installed approximately 100 m from the bridge target and anchored to a concrete wall to minimize the movement of cameras as shown in Figure 3b.

One camera was for measuring the deflection of the bridge and focused on a target installed at the web of the main arch crown. Figure 3c shows an example of an image captured by the camera. A preliminary measurement showed that the concrete wall at the slope showed some movement along with temperature change, which generated a large amount of noise during the vision measurement. As the target is 100 m from the camera, only 0.03 degree of vertical rotation of the camera can result in 50 mm of vertical deflection in the bridge. Therefore, even a small movement of the camera should be carefully corrected.

The other camera, which was for correcting the movement of the camera system, focused on a sign board installed at a building as a fixed reference point. Figure 3d shows a sign board image from the camera. If the concrete wall is displaced, the image of the fixed reference point from the building sign moves and the deflection from the first camera can be corrected by using this correcting movement. The algorithm used for the correction procedure is given in the previous study [27].

Second, the bridge temperatures were measured by using the Resistance Temperature Detector (RTD) in Figure 3e, which can measure temperatures in the range from −50 to 100 degrees Celsius with a resolution of 0.5 degrees Celsius. The RTD sensor was installed on the inner side of the center span to avoid direct exposure to sunlight.

Using the measurement systems, the vertical deflection and the bridge temperature were measured every 30 min for 4.5 months, from 15 July to 27 November 2016. Table 1 provides part of the results including the time, temperature, and deflection, and these were used as training data in this example. In Table 1, the time index is given in units of one day. Thus, it starts at 0.2500, which means 6:00 a.m. on 15 July 2016, and ends at 135.7292, which means the measurement was conducted for 135 days (i.e., 4.5 months). The positive and negative signs of the vertical deflection signify upward and downward deflection, respectively.

**Figure 4:**
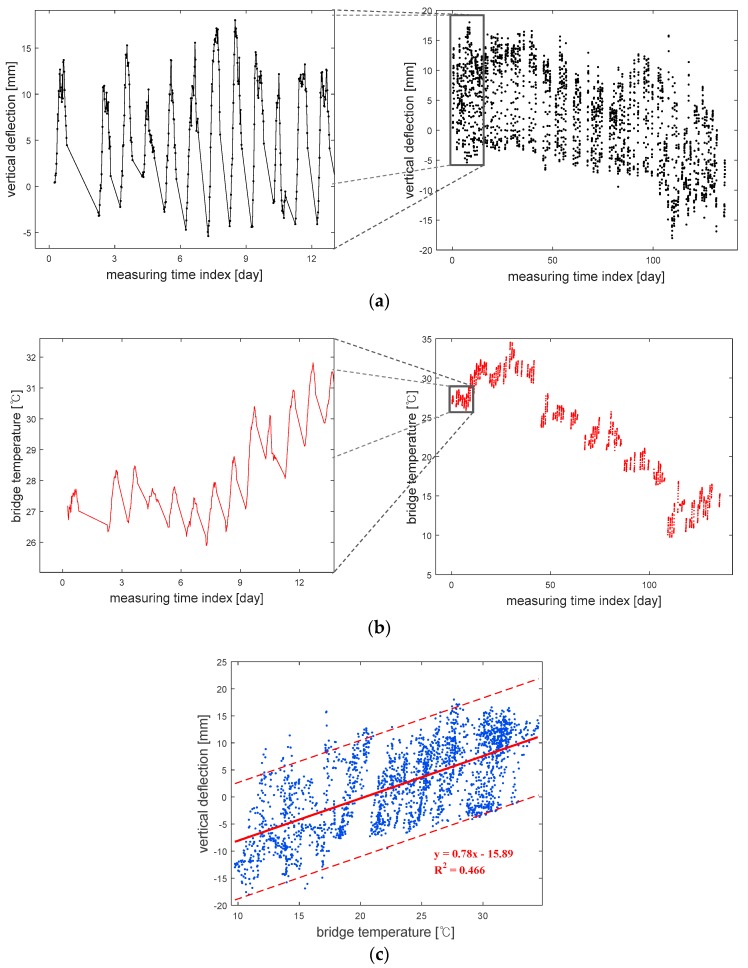
Measurement data: (**a**) vertical deflection; (**b**) bridge temperature; (**c**) bridge temperature versus vertical deflection.

The measurements that were recorded for 4.5 months produced a total of 2292 datasets of the vertical deflection and bridge temperature, and they are plotted in Figure 4a,b. Although there are some ranges without any data because the measurement devices malfunctioned, it is clearly observed that both of the vertical deflection and bridge temperature fluctuate with the same cycle within one day, which means the vertical deflection and bridge temperature are correlated. For this reason, in this example, a GP model is constructed with introducing temperature data as input.

Indeed, other factors than temperature such as creep, shrinkage, and train loads may also have an effect on the vertical deflection of a bridge. According to Nilson (2003), creep proceeds at a decreasing rate and ceases after two to five years at a final value and shrinkage continues at a decreasing rate for the first several months [46]. It was also stated by the American Concrete Institute (2008) that most of the shrinkage effects are manifested in the first year [47]. Because the example bridge was built in 2009 and the measurement was made in 2016, it was assumed in this study that the creep and shrinkage may not have a significant effect on the vertical deflection of the bridge. In addition, train load was not introduced as an important input in the example because trains only remain on the bridge for a few seconds while passing one to two times per hour and it is thus difficult to expect that a train would be passing at the moment when the bridge deflection is being measured. For these reasons, in this example, the temperature is assumed to be the most critical factor influencing the vertical deflection and is introduced as input.

Figure 4c clearly shows the positive correlation between the bridge temperature and vertical deflection. Through regression analysis, the mean and 95% prediction intervals are obtained, and the R-squared value is estimated to be 0.466, which means the temperature is an important factor relating to vertical deflection. However, it also means there can be other influential factors which were not measured in the experiment. Therefore, it is necessary to introduce probabilistic upper and lower bounds of the output, when the prediction model is built using the temperature as input data in this study.

When there is only one input as in this case, a simple regression can also give a mean equation and confidence interval for lower costs. However, a GP model can provide more flexible predictive mean and variance than regression [33,34]. Furthermore, it enables the construction of a covariance matrix with a kernel function in Equation (4) which decreases as the time interval increases. For these reasons, GP is introduced to predict the vertical deflection in this example.

For the construction of a GP model, the measuring time index and the bridge temperature are considered as input data to construct the training input matrix **X** in Equation (1), and the measured vertical deflection is used as output data in the same equation to construct the training output vector **y**. To predict the deflection based on the training data, the test input matrix **X_∗_** in Equation (2) is constructed for the time of interest and the corresponding temperature, which were obtained from the database of the Korea Meteorological Administration [48].

## 4. Analysis Results

**Figure 5:**
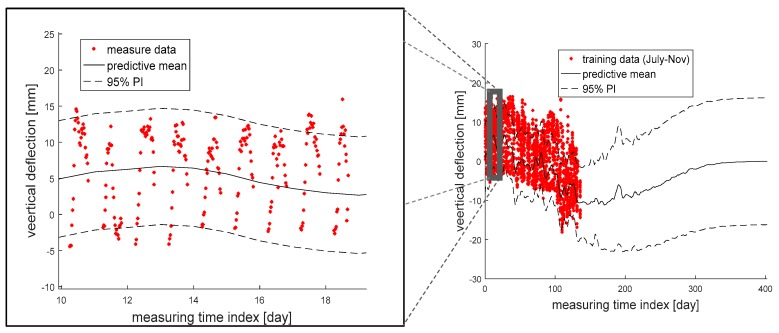
Analysis results of predictive mean and 95% prediction interval (PI) from the entire measurement data (**right**) and its zoom-in view (**left**).

The vertical deflection of Eonyang Arch Bridge was predicted by using the proposed method and the measurement data for 4.5 months, and Figure 5 shows the results of predictive mean and 95% PI. As shown in the left figure, the actual measurement data on vertical deflection fluctuate on a daily basis, and the daily deflection ranges are approximately up to ±15 mm from the average. This is mainly because the vertical deflection is correlated with the temperature which also changes on a daily basis. In addition, as shown in the right figure, another cycle with a period of hundreds of days can be found in the prediction, which is thought to be due to the seasonal changes in the temperature.

Furthermore, Guideline of Track Maintenance of Korea (2016) suggests that, in the case of the example bridge, maintenance actions need to be taken when the absolute value of vertical deflection exceeds 18 mm [5]. Figure 5 shows that the measured absolute values are mostly smaller than the threshold, but there is a small probability of violating the regulation within the next few months. Thus, it is recommended to keep monitoring the vertical deflection, even though maintenance on the example bridge does not need to be done immediately.

To test the performance of the proposed method, predictions were made using different durations of the measurement time as training data. In addition, actual measurement data that were not included in the training data were used to validate the prediction results obtained from the proposed method.

**Figure 6:**
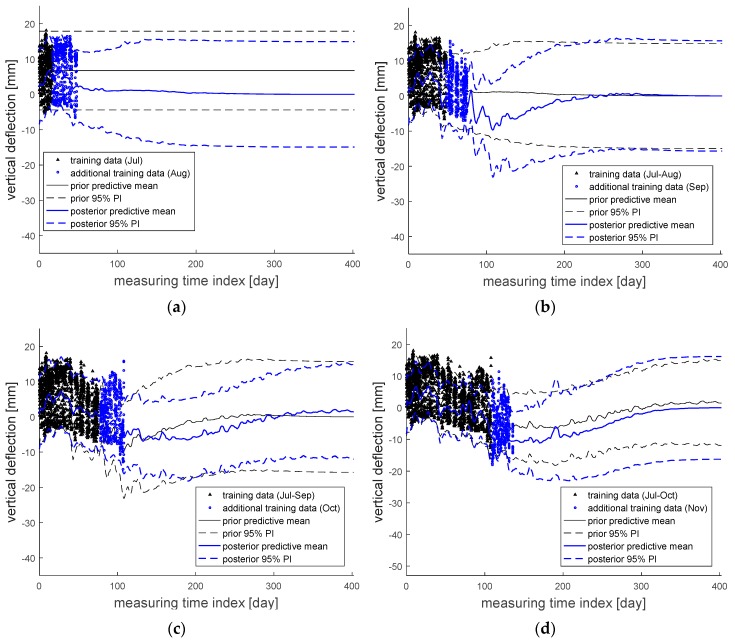
Sequentially updated predictive mean and 95% PI by adding the measurement data in: (**a**) August; (**b**) September; (**c**) October; and (**d**) November 2016.

Figure 6 shows how the predictive mean and 95% PI are updated sequentially by additional measurement data. For example, the black solid and dotted lines plotted in Figure 6a are respectively the predictive mean and 95% PI obtained from the black measurement data in July 2016 only. However, when blue additional data of the next month (i.e., August 2016) are also considered for training, the predictive mean and 95% PI are updated in blue. Likewise, the sequentially updated predictive means and 95% PIs after considering data in September, October, and November 2016 are shown in Figure 6b–d, respectively.

The models constructed in Figure 6 were checked by calculating the RMSEs with respect to the given datasets. Table 2 presents these results, and the RMSE values are estimated to be approximately five millimeters overall, with minimum and maximum values of 4.01 mm and 6.20 mm, respectively. As discussed in Section 2.2, the RMSE is calculated by comparing the predictive mean and the actual measurement results every 30 min in this example, which means the overall five millimeters represents the inherent variability of the vertical deflection which cannot be reduced. In addition, the RMSE values of five millimeters overall also mean the constructed models based on different durations of measurement time have a similar level of inherent variability.

These can be confirmed more clearly from Table 3, which shows the results of ME with respect to the given datasets utilized for training. As shown in the table, the ME values are generally close to zero, exactly in between −1.49 mm and 0.59 mm. This is because the inherent variabilities in the prediction models are canceled out (as mentioned before), which means the prediction models are close to being unbiased.

**Figure 7:**
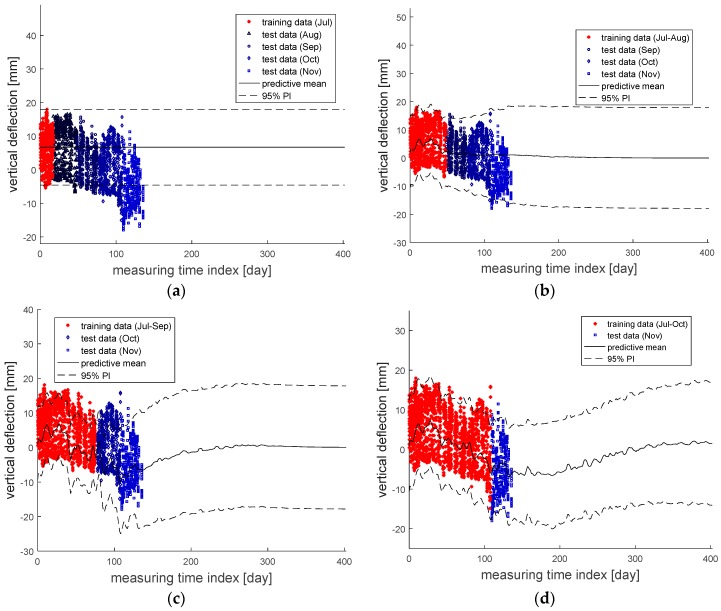
Comparison between the actual measurement data and the prediction results based on the datasets within: (**a**) July; (**b**) July to August; (**c**) July to September; (**d**) July to November.

Meanwhile, Figure 7 compares the prediction results and the actual measurement data. For example, Figure 7a shows the predictive mean and 95% PI (in black) based on the measurement data in July only and compares them with the actual measurement data obtained from August through November. It is clearly seen that the prediction accuracy gradually decreases as the moment of prediction moves farther away. However, as the duration of the measurement time for training data becomes longer, more accurate prediction models are obtained, and the prediction results become quite similar to the actual measurement data.

These points can be observed more clearly using performance assessment indices. Table 4 shows the RMSE values with respect to different sets of test data. From the table, it is first seen that the RMSE values are relatively small when the prediction results are compared with the measurement data of the subsequent months and the RMSE values increase as the moment of prediction moves farther away. Second, it is also observed that the RMSE values decrease as measurement data for a longer duration is used.

These findings are more evident with ME, where the inherent variabilities are canceled out. Table 5 provides the results of ME with respect to different sets of test data. As shown in the table, although the model that was constructed based only on the measurement data in July may seem acceptable for August with 2.36 mm, the ME values continue increasing as the moment for prediction is taken farther away from the measurement period (i.e., July). However, whenever an additional dataset is added to the training data, the absolute ME value generally decreases, which means the accuracy of the prediction model improves. For example, the ME value of the prediction model constructed using the data from July to October has a small ME value of 0.49 mm, when the model is compared with the actual measurement data for November.

On the other hand, to check the results of 95% PIs, the ACE values are calculated with respect to the given dataset. Table 6 shows the ACE values with respect to different sets of training data, and it can be seen that the ACE values are close to zero overall. This means the PI of each prediction model covers the specified portion (i.e., 95% in this example) of the given data.

In addition, Table 7 presents the ACE values with respect to different sets of test data. The two facts that were already observed with RMSE and ME are clearly noticeable. First, it is seen that ACE values are relatively small when the PIs are compared with the measurement data of the subsequent months and the absolute values of ACE increase as the moment of prediction moves farther away. Second, it is also observed that the absolute values of ACE decrease as measurement data for a longer duration are used.

## 5. Conclusions

This paper proposes a probabilistic method which employs a Gaussian process to construct a model to predict the vertical deflection of a railway arch bridge based on actual computer vision-measured data. To deal with the sources of uncertainty which may cause errors in a prediction model, a Gaussian process is modeled with multiple kernels and hyperparameters. Once the hyperparameters are identified through the Gaussian process regression using training data, the proposed method provides the predictive mean and 95% prediction interval of the vertical deflection of the target bridge. The proposed method was tested by applying it to Eonyang Arch Bridge, which is a railway bridge operated for high-speed trains in South Korea. The corresponding analysis results showed that as additional training data were introduced, both the predictive mean and predictive interval could be updated. In addition, the analysis results of the predictive mean and 95% PI obtained from the proposed method showed good agreement with the actual measurement data on the vertical deflection of the example bridge, and it was shown that the prediction results could be utilized for decision-making on railway bridge maintenance.

## Figures and Tables

**Table 1:** Part of the measurement data on Eonyang Arch Bridge.

Training Input Data			Training Output Data
Measuring Time	Measuring Time Index (day)	Bridge Temperature (°C)	Vertical Deflection (mm)
15 July 2016, 6:00	0.2500	21.45	1.195
15 July 2016, 6:30	0.2708	23.59	2.455
15 July 2016, 7:00	0.2917	25.73	3.565
...	...	...	...
27 November 2016, 16:00	135.6875	9.55	−6.127
27 November 2016, 16:30	135.7083	9.26	−6.638
27 November 2016, 17:00	135.7292	8.69	−7.300

**Table 2:** RMSE values with respect to different sets of training data.

Given Training Data	Model of July (mm)	Model of July–August (mm)	Model of July–September (mm)	Model of July–October (mm)	Model of July–November (mm)
July 2016	4.01	4.09	4.60	4.28	4.71
August 2016	-	5.43	5.58	5.39	5.66
September 2016	-	-	4.78	4.24	4.21
October 2016	-	-	-	5.89	6.2
November 2016	-	-	-	-	4.49

**Table 3:** ME values with respect to different sets of training data.

Given Training Data	Model of July (mm)	Model of July–August (mm)	Model of July–September (mm)	Model of July–October (mm)	Model of July–November (mm)
July 2016	−0.84	−1.00	−1.04	−1.09	−0.81
August 2016	-	0.29	−0.67	0.56	−0.93
September 2016	-	-	−0.82	0.59	−0.26
October 2016	-	-	-	−1.40	−1.49
November 2016	-	-	-	-	−1.41

**Table 4:** Root-mean-square error (RMSE) values with respect to different sets of test data.

Given Test Data	Model of July (mm)	Model of July–August (mm)	Model of July–September (mm)	Model of July–October (mm)	Model of July–November (mm)
July 2016	-	-	-	-	-
August 2016	6.82	-	-	-	-
September 2016	7.55	3.98	-	-	-
October 2016	7.97	5.27	4.29	-	-
November 2016	12.28	7.06	3.35	3.42	-

**Table 5:** Mean-error (ME) values with respect to different sets of test data.

Given Test Data	Model of July (mm)	Model of July–August (mm)	Model of July–September (mm)	Model of July–October (mm)	Model of July–November (mm)
July 2016	-	-	-	-	-
August 2016	2.36	-	-	-	-
September 2016	6.76	1.73	-	-	-
October 2016	5.94	0.37	−1.47	-	-
November 2016	11.82	6.22	−1.66	0.49	-

**Table 6:** Average coverage error (ACE) values with respect to different sets of training data.

Given Training Data	Model of July (mm)	Model of July–August (mm)	Model of July–September (mm)	Model of July–October (mm)	Model of July–November (mm)
July 2016	−0.0411	0.0170	−0.0165	0.0125	−0.0188
August 2016	-	−0.0045	0.0285	−0.0293	0.0120
September 2016	-	-	0.0104	−0.0379	−0.0065
October 2016	-	-	-	0.0474	0.0460
November 2016	-	-	-	-	−0.0197

**Table 7:** ACE values with respect to the test datasets.

Given Test Data	Model of July (mm)	Model of July–August (mm)	Model of July–September (mm)	Model of July–October (mm)	Model of July–November (mm)
July 2016	-	-	-	-	-
August 2016	−0.0335	-	-	-	-
September 2016	0.0273	−0.0258	-	-	-
October 2016	0.2248	−0.0397	0.0460	-	-
November 2016	0.5864	0.0482	−0.0478	−0.0284	-
